# Nonfatal Occupational Injury Rates and Musculoskeletal Symptoms among Housekeeping Employees of a Hospital in Texas

**DOI:** 10.1155/2011/382510

**Published:** 2011-06-05

**Authors:** Kirtigandha Salwe, Shrawan Kumar, Joyce Hood

**Affiliations:** ^1^Osteopathic Manipulative Medicine Department, University of North Texas Health Science Center, 3500 Camp Bowie Boulevard, Room CBH 431, Fort Worth, TX 76107, USA; ^2^School of Public Health, University of North Texas Health Science Center, 3500 Camp Bowie Boulevard, Fort Worth, TX 76107-2699, USA

## Abstract

*Objectives*. To determine the prevalence of musculoskeletal disorders in hospital cleaners. *Methods*. Injury data on all hospital employees were extracted from occupational health records and compared. Additionally an interview-based modified Nordic Questionnaire (response rate 98.14%) was conducted. *Results*. The mean total injury rate for cleaners was 35.9 per 100 full-time equivalent (FTE), while that for other employees was 13.64 per 100 FTE. Slips/trips/falls and MMH contributed 4.39 and 2.37 per 100 FTE among cleaners and rest of the hospital employees, respectively. The most common type of injury was strain while the most common cause of injury was a striking object. *Conclusion*. The cleaners have higher injury rates and morbidity as compared to other employees of the hospital. The lower back was most commonly affected.

## 1. Introduction

In United States, about 595,800 establishments make up the healthcare industry. According to the American Hospital Association, in 2009, there were over 11,000 hospitals in the United States [1]. About 21 percent of hospital jobs are in service occupations, such as nursing, psychiatric, and home health aides, or cleaners [2]. The International Labour Organization's (ILO) key indicators of the labor market suggest that wages for work such as performed by cleaners is low in comparison to most other occupations [3]. More specifically, employment statistics on jobs in the US show that the mean annual wage estimates of housekeepers are approximately one-half of the mean annual wage estimates for all occupations [4]. In 2008, the total number of nonfatal occupational injuries in hospitals (private industry, state government, and local government) was 323,200 [5]. The rate of occupational injuries and illnesses among cleaners was 3.9 per 100 full-time workers [6]. Such injuries and illnesses affect the job performance of the cleaners thus affecting their efficiency. Due to the reduced efficiency and absenteeism, the employers have to incur losses and the treatment and rehabilitation of these employees are costly to society. This also leads to loss of wages to the cleaners due to absenteeism. Many of the cleaners develop conditions like arthritis, tendonitis, carpel tunnel syndrome, and other musculoskeletal conditions. The tasks performed by cleaners are labor intensive and most of the cleaners have to work under time constraints increasing the physical and mental stress. A study carried out in four countries of the European Union showed that the prevalence of health problems like musculoskeletal problems, skin problems, and psychosomatic disorders among cleaners is high compared to the average wage earners in general [7]. Occupational physical risk factors responsible for musculoskeletal morbidity include repetitive work, working with hands above shoulder height or below knee height, carrying heavy loads, and operating vibrating tools [8, 9]. All of the above-mentioned risk factors are prevalent in cleaners and may therefore contribute toward musculoskeletal morbidity in the cleaning occupation. The Bureau of Labor Statistics provides the number of nonfatal occupational injuries and illnesses for cleaners; however, there is no record of these among cleaners specific to hospitals. It is apparent from the review of literature that little work has been done on the cleaning workers specific to hospitals. A large number of studies have investigated the association of musculoskeletal disorders and physical workload [10–12]. Most studies have suggested the evidence of physical work factors as determinants of musculoskeletal disorders of the upper extremity and the low back [10, 13, 14]. Association between physically demanding work tasks and disorders of the lower extremity is evident in some studies [15–17]. However, there is little evidence of similar studies exclusively for hospital cleaners. Therefore, the specific objectives of this study were to (a) determine the rates of nonfatal occupational injury and illness in hospital cleaners in one hospital in Texas and (b) investigate the prevalence of musculoskeletal symptoms in this population in one Texas hospital. The focus of this study is on injuries related to slip/trip/fall, material handling, and work-related musculoskeletal aches, pains and discomfort. This study explores information about incidence rates among hospital cleaners categorized by various factors such as type of injury and source of injury. It investigated prevalence rate of musculoskeletal symptoms among the hospital cleaners.

## 2. Methods

### 2.1. Study Sample

 The study sample comprised of 106 of 108 housekeeping employees of a hospital located in Texas, United States giving a response rate of 98.14%. The mean age of the sample was 46.36 years (SD 13.89 years), and median age was 48.5 years. The age of the workers ranged between 19 and 72 years. The cleaners worked for an average of 8 h per day in one of three shifts. 51% worked during the first shift (7 am to 3 pm), 35% worked during the second shift (3 pm to 11 pm), and 14% worked during the third shift (11 pm to 7 am). The number of years they worked in cleaning jobs ranged from 1 year to 40 years with a mean of 14.2 years (SD 9.84 years).

### 2.2. Tasks

 The cleaners perform repetitive cleaning of assigned patient rooms, dismissals, units, X-ray rooms, surgical unit, recovery rooms, all offices, and other areas using standard cleaning supplies and disinfectants. As part of their job profile, they spend 40% of their time cleaning patient rooms, clinics, and offices, 25% of their time emptying trash and linen, and 10% of their time vacuuming and shampooing carpets. They spend 5% of their time on mopping floors, cleaning furniture, refinishing floors, moving furniture, and responding to hospital emergency drills, respectively. The cleaners carry supplies of linens, towels, toilet items, and cleaning materials, using carts and keep storage areas clean and tidy and carts well stocked. Cleaning patient rooms, clinics, and offices involves pushing/pulling beds, turning the mattress, moving furniture, dusting furniture, sweeping and mopping the floor. Moving trash involves lifting trash bags and dumping them into a cart, walking long distances pushing the cart to the dump station, and emptying the trash into the electric dumpster. Cleaning bathrooms involves bending over and stooping to clean toilets and tubs, washing sinks, mopping floors, refilling soap, and loading toilet paper. Sweeping floor is done with a broom with a long handle and dust pan. The ceramic tiles and vinyl flooring materials are cleaned using mops and washcloths. Water from the mop is squeezed by wringing the washcloths using a wringer on the mop bucket. Washing windows, walls, ceilings, woodwork, and waxing and polishing as necessary is also done. Cleaning rugs, carpets, upholstered furniture, and/or draperies, using vacuum cleaners and/or shampooers are common housekeeping tasks. The cleaners are also required to prepare rooms for meetings, arrange media equipment, and furniture for social or business functions in auditoria of the hospital. The linen room has three employees who sort, count, fold, and store linen in closets; they also move linen carts to various units of the hospital.

### 2.3. Data Collection and Questionnaire

 The study was conducted in two parts. In the first part, employee injury and illness reports were obtained from the Occupational Health Services of the hospital. The report consisted of information about 117 incidents among cleaners from 2004 to 2008. 

 The second part consisted of a cross-sectional survey using an interview-based modified Nordic Musculoskeletal Questionnaire [18] to study the extent of musculoskeletal symptoms of different body parts among the housekeeping employees. The study was approved by the university and the hospital Institutional Review Board. Informed consent was obtained from the participants in English and in Spanish where applicable. Responses to the questionnaire were noted. The questionnaire comprised of four sections: demographic information, information on pain and discomfort, types of work tasks, and work organization. The demographic information included age, gender, height, body weight, number of hours worked each day, areas of the hospital assigned to clean, number of years working in the hospital, and total number of years in the cleaning work. Subjective complaints of pain and discomfort from neck, shoulders, elbows, wrist/hands, upper back, lower back, hips/thighs/buttocks, knees, and ankles/feet during the last 12 months were obtained. The employees who reported pain or discomfort were asked to identify the painful body parts on a body map showing the body regions and the level of pain on a scale of 0 to 10 ranging from “no pain at all” to “worst imaginable pain”. The level of pain was categorized as mild (score: 1 to 3), moderate (score: 4 to 7), and severe (score: 8 to 10). The third section included questions on buffing, vacuuming, lifting equipment, mopping floors, carrying garbage and linen, cleaning bathrooms, making beds, moving furniture, carrying heavy loads, and working with the back, neck, or arms in awkward postures. In the study, the employees were asked about the frequency of their work tasks. The answers were categorized into three classes (1 = frequently, 2 = sometimes, 3 = never). The work organization section asked participants if they had to work fast, work intensively and if they had enough time to do their work.

### 2.4. Data Analysis

Participants were grouped as per gender, age, number of years in cleaning occupation, race, body mass index, and shift work. The prevalence of musculoskeletal aches, pains and discomfort among cleaners in the past 12 months in association to the above-mentioned demographic variables and selected body parts was calculated. Incident rates among the housekeeping staff and the rest of the hospital staff were extracted. The incidence rates in this sample represent the number of injuries per 100 full-time employees (FTE). The incidence rates were calculated as (N/H) ×200,000 where *N* = number of injuries and illness, H = total hours worked by all employees during period covered, and 200,000 = represents the equivalent of 100 employees working 40 h/week, 50 weeks/year. The incidence rates for all injuries, for injuries due to slip/trip/fall, and injuries due to material handling among cleaners and rest of the hospital staff were compared. The 117 incidents among cleaners were categorized as strains, contusion/abrasion, laceration/scratch, puncture wound, chemical exposure, others and the number of cases in each category was calculated. These incidents were further classified by cause of injury. The frequency of execution of different tasks performed by housekeepers was extracted thus calculating the percentage of housekeepers performing tasks frequently, sometimes, and never.

## 3. Results

 Background demographic characteristics and the prevalence of musculoskeletal pain for the study population are shown in Table 1. Female cleaners had a higher prevalence of musculoskeletal symptoms in the last 12 months than male cleaners. Black cleaners had a higher prevalence of pain followed by Hispanics. Cleaners in age group from 39 to 58 years, housekeepers working in housekeeping jobs for over 31 years, housekeepers with Body Mass Index (BMI) less than 18.5, and those working in the first shift had the highest prevalence of musculoskeletal pain in the last 12 months. This being a hospital setting, the workload for cleaners during the first shift is a lot more as compared to other shifts. This could be attributed to the influx of patient admissions during the first shift and the cleaning of surgical units and other units where the workload is highest in the morning hours. The incidence rate for total occupational injuries in cleaners for each year from 2004 to 2008 was significantly higher than that for all other employees of the hospital with the highest incidence rate during 2005 (75.18 per 100 FTE) (Table 2). 

The drop in the incidence rates in the subsequent years can be attributed to preventive measures that were taken by the Occupational Health Services of the hospital to address the high incidence rate. Mandatory slip resistant footwear, lowering of threshold plates for those areas where carpet to tile transitions (to reduce pushing/pulling injuries), ergonomic mops, soiled laundry receptacles that emptied from the bottom, department-specific education about ergonomics, and enforcement of use of appropriate personal protective equipment were some of the measures taken to reduce the injuries and illnesses among cleaners. Nursing services were also included in training so that laundry hampers were not overfilled, which left an MSD hazard for the cleaners. The five-year incidence rate of total injuries for the housekeeping employees was found to be 28.32 per 100 FTE and that for rest of the employees of the hospital was 13.54 per 100 FTE (*P* < .05). The incidence rate of slips/trips/falls among the housekeeping employees and rest of the employees of the hospital was 4.39 per 100 FTE and 2.37 per 100 FTE, respectively (*P* < .05). The incidence rate of injury due to material handling in the housekeeping employees and rest of the employees of the hospital was 5.45 per 100 FTE and 1.08 per 100 FTE, respectively (*P* < .05) (Table 2). The most common type of injury among housekeepers was due to strains followed by contusion/abrasion (Table 3). 

The most common cause of injury was being struck by an object (Table 3). Symptoms in the lower back were most prevalent (49%) followed by the right wrist (43%), ankle, left knee, left wrist (35% each), right knee (34%), right shoulder (25%), and other selected body parts (Table 4). 

Similarly, current pain and discomfort was reported by 42% of the cleaners. In this sample, 82% of those who complained of pain in the last 12 months reported that the pain was related to work. 4% of the participants complained of mild pain in the wrists, whereas 16% had moderate pain and 11% had severe pain in the wrists. The frequency of execution of different tasks by housekeepers was expressed as percent (Table 5).

 The prevalence of musculoskeletal pain was highest in participants who worked with the back in the awkward posture (65%), followed by employees who worked with their arms and neck in the awkward posture, respectively (64%). The employees who cleaned bathrooms had a prevalence of 64%, followed by those who mopped floor and carried/emptied garbage (63%). The prevalence of musculoskeletal pain in the participants who carried heavy loads, made beds, moved furniture, and used the vacuum ranged between 61% and 63% (Table 6).

## 4. Discussion

 In order to determine the number and incidence rate of nonfatal occupational injuries and illnesses among hospital housekeepers, the authors used the injury data from the housekeeping employees of a hospital to extrapolate possible number of injuries in this occupation in healthcare industry. As per the 2008 statistics, in the United States, cleaners held about 1.5 million jobs. Hospitals employed about 17 percent of these workers [2], thus making it approximately 255 thousand cleaners in hospitals. In 2008, there were 22 injuries among 108 housekeeping employees studied. Considering approximately 255,000 cleaners in the hospitals, in 2008, cleaners would have over 52,000 injuries. Since musculoskeletal disorders (MSDs) affect a significant proportion of the workforce, they are a major problem in several economic activity sectors in industrialized countries [19]. Occupational injuries in the US cost employers about $200 billion annually. This includes an estimated $155 billion for occupational injuries [20]. In this study, for 108 housekeeping employees the cost incurred by the hospital in 2008 for occupational injuries and illnesses was $127,955, thus extrapolation of this rate makes it approximately over $302 million for claim costs for all the hospital cleaners in US in 2008. The true economic burden of work-related musculoskeletal disorders is likely to be even greater because many cases are not reported to the workers' compensation system. A study of individuals diagnosed with work-related musculoskeletal disorders of the upper extremity, neck, or low back reported that only 25% filed a workers' compensation claim [21]. A population survey in Connecticut, estimated that one-tenth of working-age adults have a work-related musculoskeletal condition, but only 10% of these workers file compensation claims [22]. 

 The cleaners in this study had higher rates of injury due to slip/trip/fall and material handling. These findings are similar to other studies. Slips and falls were reported as the leading cause of death in the workplace and source of more than 20% of all disabling injuries [23]. Occupational injuries associated with slip and fall accidents pose a significant problem to industry both in terms of human suffering and economic losses. In terms of the labor costs, over one-quarter of overall fall-related injuries resulted in 31 days or more workdays being lost, costing US economies nearly $10 billion/year [24]. In the self-reported work-related illness in 1995 survey, musculoskeletal disorders were by far the most commonly reported class of work-related illness, and it was estimated that 52% of the 1.2 million reported a work-related musculoskeletal disorder due to manual handling associated with their job [25]. Manual handling is not only one of the frequent workplace activities, but is also one of the most common causes of workplace injury [26].

 The number of sprains and strains for all private industries put together (416,620 in 2008) was nearly 4 times the number of bruises and contusions (93,650 in 2008), the second highest type of injury of interest to ergonomists [27]. Injuries to the back were the highest category of injuries in the service occupations (about 31%). All the other industries had at least 23% injuries being back injuries [28]. These findings are similar to those noted in this study. In terms of their contribution to total workers' compensation costs, at the workplace, back problems are the single most costly injury. In 1992, back cases represented 24% of US workers' compensation claims and 31% of costs [29].

 After low back pain, the next common body part affected in this sample was the wrist. In a similar study it was found that wrist/hand, elbow and knee pain were more prevalent among cleaners; neck, shoulder, low back, hip, and ankle pain/discomfort was similar to other referent groups, while upper back pain was reported less by cleaners [30]. An occupational injury profile of the American industry states that upper extremity injuries, including injuries to the wrists, the hands, and the fingers, were the next highest category of injuries after back injuries. This correlates to the presence of pain in the back and wrist in descending order in the participants of this study. 

 The prevalence of musculoskeletal pain and the trend in incidence rates in this sample were similar to the prevalence in other studies [24, 30, 31]. The lower back was most commonly affected. This can be explained by the high prevalence of pain among participants who worked with their back in an awkward posture. As it is seen in other studies, it is evident from this study too that the cleaning occupation has a high prevalence of musculoskeletal pain which can predispose the cleaners to musculoskeletal disorders and injuries. Several studies have indicated the various risk factors for musculoskeletal ill-health, high workload, repetitive motion, speed and intensity of work, and weak organizational strategies to name a few [30]. 

There is evidence that exposure to each of these ergonomic factors causes MSDs in one or more body regions: repetitive upper extremity motion patterns; forceful exertions, nonneutral body postures; and vibration. The risk is especially pronounced when a job includes exposure to a combination of two or more of these risk factors [32]. All authors found that the most significant risk factors associated with the physical work of cleaners are static muscle loads (much of which involves bending and twisting of the back) and repetitive movements of the arms and hands using a high output of force. These types of prolonged static and repetitive muscle activities cause muscle fatigue and may lead to musculoskeletal disorders [33]. A sizable proportion of MSDs among exposed workers are preventable, and protective action is both warranted and necessary [32].

 Concentrating only on physical ergonomic factors like equipment design, reduction in forces, and improvement in postures may not achieve as much benefits in terms of reduction in sickness rates as a more holistic approach would, which also takes account of work organizational risk factors [18]. A holistic approach is essential to address these issues and reduce the incidence rates of occupational injuries and illnesses. Health and safety researchers and practitioners are constantly trying to control workplace injuries by trying to understand the causal mechanisms underlying workplace injuries and by designing safer working conditions through job, equipment and workplace design, educating employees, and matching employees to jobs [28]. 

 A review by Bigos et al. [34] on preventing episodes of low back pain suggests a strong evidence that exercise programs are effective, whereas other interventions including education alone (ergonomic, back school, and stress management), back supports (back belts), and shoe inserts were not effective Daily sessions of exercise and relaxation techniques before the employee starts work will help reduce the tension in the muscles thus preparing the body for various work related postures. A study by Toivanen et al. [35] showed that regular relaxation training at the workplace to provide stress management diminished tension in the neck-shoulder region efficiently and decreased absenteeism. Inter correlations were found between the neck-shoulder tension, psychosocial factors, depression, and the absentee rate. Thus a continued application of a combination of regular exercise, relaxation training, participatory ergonomic training programs, team work, work organization changes, and effective communication between the cleaners and the supervisors may help in curbing musculoskeletal ill-health among the housekeepers. 

There are several limitations to our study. First, the findings may not be generalizable as they are not based on representative random sample drawn from hospital cleaners around the country. However, a response rate of 98.1% clearly indicates the validity of findings at that institution. Another limitation is that our study assessed work-relatedness of pain or injury (and the severity of these conditions) by self-report. Data were not available to validate doctor visits for work-related pain or workers' compensation claim reporting and acceptance rates. However, the self-report data was supported by medical or administrative records making it more reliable, and also self-reports can be a more reliable source for determining the frequency of work-related pain. The questionnaire used was interview based. The questions were answered based on the memory of the participant thus the possibility of recall bias exists. Recall may have been influenced by such factors as presence of pain or negative affect. Further research is required to study the risk factors associated with musculoskeletal pain and discomfort in each body part to develop better intervention strategies.

## Figures and Tables

**Table 1:** Demographic characteristics of the study sample.

Characteristics	*N*	%	Prevalence *N* (%)
Gender			
Males	32	30.2	14 (43.75)
Females	74	69.8	54 (72.97)
Age group			
18 to 38 yr	27	25.5	14 (51.85)
39 to 58 yr	58	54.7	40 (68.96)
>59 yr	21	19.8	14 (66.66)
Years in cleaning occupation			
0 to 10 yr	44	41.5	22 (50)
11 to 20 yr	37	34.9	25 (67.56)
21 to 30 yr	19	17.9	15 (78.94)
>31 yr	6	5.7	6 (100)
Race			
Asians	27	25.5	15 (55.55)
Blacks	30	28.3	21 (70)
Caucasians	11	10.4	7 (63.63)
Hispanics	38	35.8	25 (65.78)
Body Mass Index			
<18.5	2	1.9	2 (100)
18.5 to 24.9	32	30.2	19 (59.37)
25 to 29.9	30	28.3	19 (63.33)
>30	42	39.6	28 (66.66)
Shift			
1st shift	54	50.9	40 (74.07)
2nd shift	37	34.9	21 (56.75)
3rd shift	15	14.2	7 (46.66)

**Table 2:** Total Injuries and incidence rates among housekeepers and other employees.

	Total injuries		Total injury incidence rates	
	Other employees	Housekeepers	Other employees	Housekeepers
2004	413	27	20.1	33.28
2005	268	31	13.3	75.18
2006	286	22	13.9	29.68
2007	241	15	11.3	17.88
2008	225	22	9.6	23.54

Total	1433	117	13.64	35.91

	Slip/trip/fall Injuries		Slips/trips/falls Incidence Rate	
	Other employees	Housekeepers	Other employees	Housekeepers

2004	43	2	2.1	2.46
2005	49	6	2.44	7.77
2006	56	3	2.72	3.87
2007	47	3	2.2	3.57
2008	56	4	2.4	4.28

Total	251	18	2.372	4.39

	Material handling injuries		Material handling incidence rate	
	Other employees	Housekeepers	Other employees	Housekeepers

2004	29	2	1.41	2.46
2005	27	8	1.34	12.96
2006	19	5	0.92	6.46
2007	23	4	1.08	4.76
2008	16	3	0.68	3.22

Total	114	22	1.086	5.972

**Table 3:** Number of injury cases among housekeepers by type and cause of injury.

Type of injury	2004	2005	2006	2007	2008	Total	Percent
Strain	5	13	9	6	6	39	30
Contusion/Abrasion	8	9	5	5	7	34	29
Laceration/Scratch	4	6	4	1	2	17	15
Puncture wound	6	1	2	2	1	12	12
Chemical Exposure	1	1	1	1	4	8	6
Other	3	1	2	0	2	7	8

Total	27	31	23	15	22	117	100

Cause of injury	2004	2005	2006	2007	2008	Total	Percent

Striking Object	9	4	6	3	6	28	27
NTC/Sharp Object	6	6	4	2	2	20	17
Lifting/Moving Objects	2	8	5	5	3	23	17
STF/TRPOBJ	2	6	3	3	5	19	15
Awkward Posture	2	1	1	—	—	4	4
Chemicals	1	1	—	—	2	4	3
Other/EQUMAL	5	5	2	3	4	19	17

Total	27	31	21	16	22	117	100

NTC:Needle trash cleaning;

STF:slips/trips/falls;

TRPOBJ:Tripping on an object;

EQUMAL: Equipment Malfuntio.

**Table 4:** Prevalence of pain in last 12 months in selected body parts.

Body part	*N*	Prevalence
Neck	11	16
Right Shoulder	17	25
Left Shoulder	14	21
Right elbow	7	10
Left elbow	1	1
Right wrist	29	43
Left wrist	24	35
Upper back	7	10
Lower Back	33	49
Right knee	23	34
Left knee	24	35
Ankle	24	35
Hips/thighs/buttocks	4	6

**Table 5:** Percentage of participants conducting tasks.

Task	Frequently		Sometimes		Never	
	*N*	%	*N*	%	*N*	%
Use buff	9	9	15	14	82	77
Use vacuum	20	19	72	68	14	13
Mop and wipe floors	92	87	11	10	3	3
Carry and empty garbage	96	91	7	7	3	3
Clean bathrooms	86	81	16	15	4	3
Make beds	71	67	23	22	12	11
Move furniture	77	73	22	21	7	7
Carry heavy loads	56	53	27	26	23	22
Work with back in awkward posture	45	43	33	31	28	26
Work with arms in awkward posture	49	46	31	30	26	25
Work with neck in awkward posture	49	46	27	26	30	28

**Table 6:** Prevalence of pain in last 12 months by tasks.

Task	*N*	Prevalence *N* (%)
Use buff	24	8 (33)
Use vacuum	92	58 (63)
Mop and wipe floors	103	65 (63)
Carry and empty garbage	103	65 (63)
Clean bathrooms	102	65 (64)
Make beds	94	59 (63)
Move furniture	99	62 (63)
Carry heavy loads	83	51 (61)
Work with back in awkward posture	78	51 (65)
Work with arms in awkward posture	80	52 (65)
Work with neck in awkward posture	76	49 (64)
